# A simple and rapid Hepatitis A Virus (HAV) titration assay based on antibiotic resistance of infected cells: evaluation of the HAV neutralization potency of human immune globulin preparations

**DOI:** 10.1186/1743-422X-5-155

**Published:** 2008-12-18

**Authors:** Krishnamurthy Konduru, Maria Luisa Virata-Theimer, Mei-ying W Yu, Gerardo G Kaplan

**Affiliations:** 1 Division of Emerging and Transfusion Transmitted Diseases, Center for Biologics Evaluation and Research, Food and Drug Administration, 29 Lincoln Drive, Bethesda, Maryland 20892, USA; 2 Division of Hematology, Center for Biologics Evaluation and Research, Food and Drug Administration, 29 Lincoln Drive, Bethesda, Maryland 20892, USA

## Abstract

**Background:**

Hepatitis A virus (HAV), the causative agent of acute hepatitis in humans, is an atypical Picornaviridae that grows poorly in cell culture. HAV titrations are laborious and time-consuming because the virus in general does not cause cytopathic effect and is detected by immunochemical or molecular probes. Simple HAV titration assays could be developed using currently available viral construct containing selectable markers.

**Results:**

We developed an antibiotic resistance titration assay (ARTA) based on the infection of human hepatoma cells with a wild type HAV construct containing a blasticidin (Bsd) resistance gene. Human hepatoma cells infected with the HAV-Bsd construct survived selection with 2 μg/ml of blasticidin whereas uninfected cells died within a few days. At 8 days postinfection, the color of the pH indicator phenol red in cell culture media correlated with the presence of HAV-Bsd-infected blasticidin-resistant cells: an orange-to-yellow color indicated the presence of growing cells whereas a pink-to-purple color indicated that the cells were dead. HAV-Bsd titers were determined by an endpoint dilution assay based on the color of the cell culture medium scoring orange-to-yellow wells as positive and pink-to-purple wells as negative for HAV. As a proof-of-concept, we used the ARTA to evaluate the HAV neutralization potency of two commercially available human immune globulin (IG) preparations and a WHO International Standard for anti-HAV. The three IG preparations contained comparable levels of anti-HAV antibodies that neutralized approximately 1.5 log of HAV-Bsd. Similar neutralization results were obtained in the absence of blasticidin by an endpoint dilution ELISA at 2 weeks postinfection.

**Conclusion:**

The ARTA is a simple and rapid method to determine HAV titers without using HAV-specific probes. We determined the HAV neutralization potency of human IG preparations in 8 days by ARTA compared to the 14 days required by the endpoint dilution ELISA. The ARTA reduced the labour, time, and cost of HAV titrations making it suitable for high throughput screening of sera and antivirals, determination of anti-HAV antibodies in human immune globulin preparations, and research applications that involve the routine evaluation of HAV titers.

## Background

Hepatitis A Virus (HAV), a Picornaviridae that causes acute hepatitis in humans, is a significant public health problem in developing nations with approximately 1.4 million new infections per year [1]. The virus is mainly transmitted via the fecal-oral route, either from person to person or by ingestion of contaminated food and water. Community wide outbreaks can result from the consumption of oysters and mussels harvested from contaminated waters, fresh produce from contaminated water-irrigated fields, and food prepared by infected handlers [2-4]. For example, a recent HAV outbreak originated from contaminated green onions resulted in over 600 infection cases and 3 deaths [5]. Hepatitis A is an age-dependent disease, and children 6 year old and younger in general develop a subclinical form of the disease. Older children and adults develop a more severe form of hepatitis A, which in some rare instances can result in fulminant hepatitis. In developing countries, water- and food-borne HAV infections are common during childhood, which induces life-long immunity.

The overall incidence of HAV has decreased in recent years in the United States and Europe [6,7] but the proportion of travel-related cases has increased in the United States. HAV vaccination and immune globulin (IG) are recommended for international travellers who plan to visit countries that are considered intermediate to high endemic zones for HAV infection [7,8]. IG is recommended in addition to vaccination for elderly persons who are immunocompromised, have chronic liver disease, or have chronic medical conditions and are travelling to endemic zones. HAV vaccine does not prevent infection if administered three or more weeks post virus infection, but protection is conferred by administration of IG two weeks after exposure to the virus [9,10]. It has recently been shown that both HAV vaccine and IG are similarly effective for post-exposure prophylaxis within 2 weeks of the exposure to HAV [11]. IG preparations are derived from pools of plasma from human donors. Anti-HAV antibody levels vary among different lots of IG preparations [12]. HAV vaccinated donors tend to have 10–50 fold lower anti-HAV titers than donors who were naturally infected with HAV [13].

HAV grows poorly in cell culture and in general does not induce cytopathic effect (CPE). Cytopathic strains of HAV have been isolated but CPE takes a long time to develop, the plaques are difficult to visualize, and CPE is dependent on the multiplicity of infection [14,15]. Modified HAV plaque assays that detect HAV antigen in fixed cells have been developed but are time-consuming and laborious [15-17]. ELISA-based endpoint dilution assays to titrate HAV are simple to perform but require 2 weeks of incubation to detect antigen at the higher dilutions [18-23]. We reported previously that the insertion of a blasticidin resistance (Bsd) gene into the genome of wild type (wt) HAV allowed the selection of a cell line with enhanced susceptibility to wt HAV infection [24]. In the present study, we used the HAV-Bsd construct to develop a rapid and simple titration assay based on the selection of blasticidin-resistant cells, and used this assay to evaluate the HAV neutralization potency of commercially available human IG preparations.

## Results

### Titration of HAV by the antibiotic resistance titration assay (ARTA)

**Figure 1:**
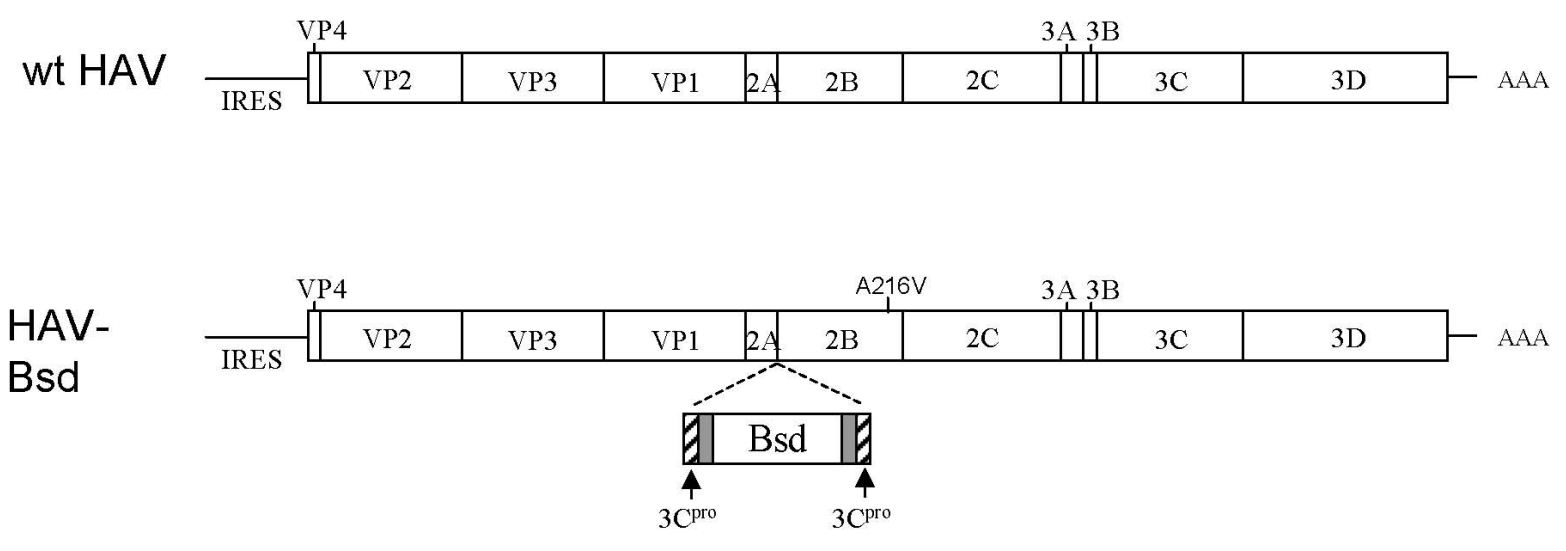
**Schematic representation of the genome of the HAV-Bsd**. The infectious cDNA of wild type HM-175 strain of HAV (wt HAV) containing a A216V amino acid substitution in the 2B protein was used as the background to construct HAV-Bsd. The blasticidin resistance (Bsd) gene coding for the blasticidin deaminase was inserted into the 2A-2B junction of the infectious cDNA of HAV [24]. The Bsd gene was flanked by three G residues (gray box) and an HAV protease 3C^pro ^cleavage sites (dashed box) at each end. The three G residues form a hinge that facilitates the processing of the adjacent 3C^pro ^cleavage site and the release the blasticidin deaminase from the HAV polyprotein.

**Figure 2:**
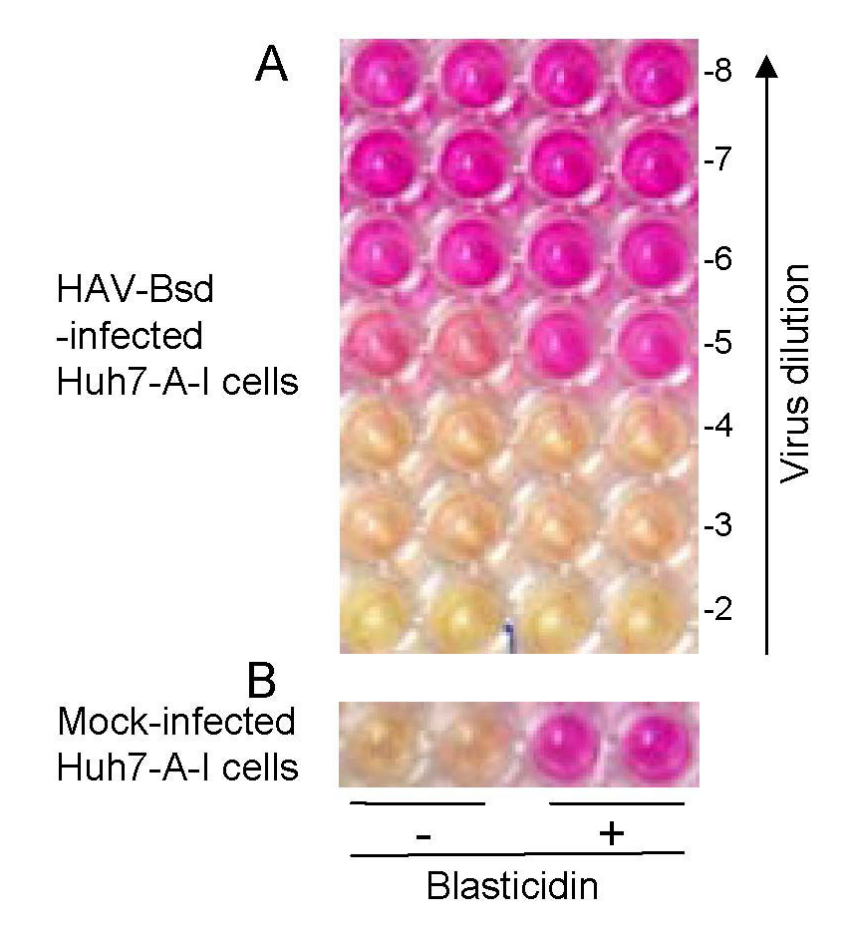
**HAV titration by ARTA**. (**A**) Huh7-A-I cells in 96-well plates were infected with ten-fold serial dilutions of HAV-Bsd and selected with 2 μg/ml blasticidin. Four wells were infected with each virus dilution. Plates were incubated for 8 days at 35°C and analyzed by the naked eye and under the microscope. A color photograph of the plate was taken a few minutes after removing the plate from the CO_2 _incubator. The color of the cell culture medium of all wells at dilution -2 to -4 and a single well at dilution -5 was orange-to-yellow, and these wells contained live cells as confirmed by microscope examination (not shown). The cell culture media color of three wells at dilution -5 and all the wells at dilutions -6 to -8 was pink to purple, and these wells contained dead cells as assessed under the microscope. The yellow and orange wells were considered positive for HAV-Bsd. (**B**) Four control wells containing Huh7-A-I cells were mock-infected and treated with 2 μg/ml blasticidin (+) or cell culture medium (-). The cells treated with blasticidin died and the cell culture media color turned pink-to-purple. The cells that did not receive the antibiotic were healthy and the color of the cell culture media was orange-to-yellow.

To develop a simple and rapid titration method, we used a wild type HAV construct containing a Bsd resistance gene inserted into the 2A-2B junction [24]. In addition to this selectable marker, this HAV-Bsd construct contained an Ala-to-Val substitution at amino acid 216 of the 2B protein (Figure 1) that enhanced its growth in cell culture but did not attenuate the virus [25]. HAV-Bsd grew efficiently in Huh7-A-I cells, a clone of human hepatoma Huh7 cells that supports the stable growth of wt HAV [24]. Huh7-A-I cells infected with HAV-Bsd were resistant to a low concentration of blasticidin (2 mg/ml) that killed uninfected cells. Since the HAV-Bsd-infected Huh7-A-I cells, but not the uninfected cells, continued to metabolize and acidify the cell culture media in the presence of blasticidin, the color of the pH indicator in the cell culture media could be used as a surrogate marker for the presence of HAV-Bsd-infected cells. In an endpoint dilution assay using multiwell plates, the color of the cell culture media could be used to identify HAV-Bsd positive and negative wells without further processing of the plates. To test our hypothesis, HAV-Bsd was titrated on 96-well plates containing Huh7-A-I cells, and incubated at 35°C for 8 days in the presence of 2 μg/ml blasticidin (Figure 2). Examination of the 96-well plates under the microscope showed that the orange-and-yellow wells contained healthy cells whereas the pink and purple wells contained rounded and detached dead cells (data not shown). This direct correlation between the color of the cell culture media and the presence or absence of live HAV-Bsd infected cells was used to calculate viral titers using the Reed and Muench method [26] and the ID50 computer program.

### Evaluation of total anti-HAV antibodies in human IG preparations

**Figure 3:**
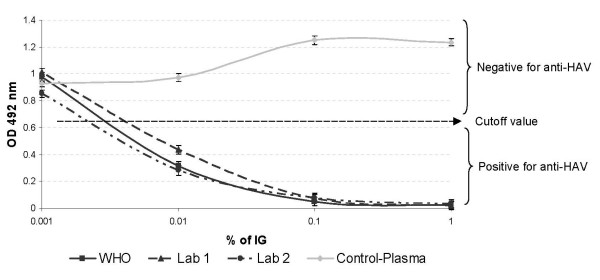
**Analysis of anti-HAV antibodies in IG preprations**. Semi-quantitative analysis of anti-HAV antibodies in the immune globulin preparations was performed with the HAVAB EIA kit. Ten-fold serial dilutions of the Lab 1, Lab 2, and WHO standard IG preparations in complete medium were tested in duplicates for the presence of anti-HAV antibodies. The negative control plasma control was also tested. The cutoff value was obtained from control reagents included with the HAVAB EIA kit. In this competitive assay, values above and below the cutoff were considered negative and positive, respectively. The means of duplicate determinations are plotted and the standard deviations are shown as bars.

To determine whether the ARTA could be used to evaluate the HAV neutralization potency of human IG preparations, we first analyzed the levels of anti-HAV antibodies present in two commercially available human IG preparations from different manufacturers (Lab 1 and Lab 2). The 1^st ^WHO International Standard for Hepatitis A Immunoglobulin (reconstituted as 100 IU/ml) was used as a positive control IG preparation, and an anti-HAV antibody-negative human plasma donation containing ~1% IgG was used as a negative control. All the IG preparation stocks contained approximately 16% IgG solutions. Presence of anti-HAV antibodies in ten-fold dilutions of the IG preparations was evaluated using the HAVAB EIA kit (Figure 3), a competitive assay with a cutoff value under which samples are considered positive for anti-HAV antibodies. All dilutions of the Lab 1, Lab 2, and the WHO Standard IG preparations reacted similarly in the HAVAB EIA test indicating that these IG preparations contained comparable levels of anti-HAV antibodies. The absorbance values of all the dilutions of the negative control plasma were above the cutoff indicating the absence of anti-HAV antibodies.

### Evaluation of anti-HAV neutralizing antibodies in the human IG preparations by ARTA

**Figure 4:**
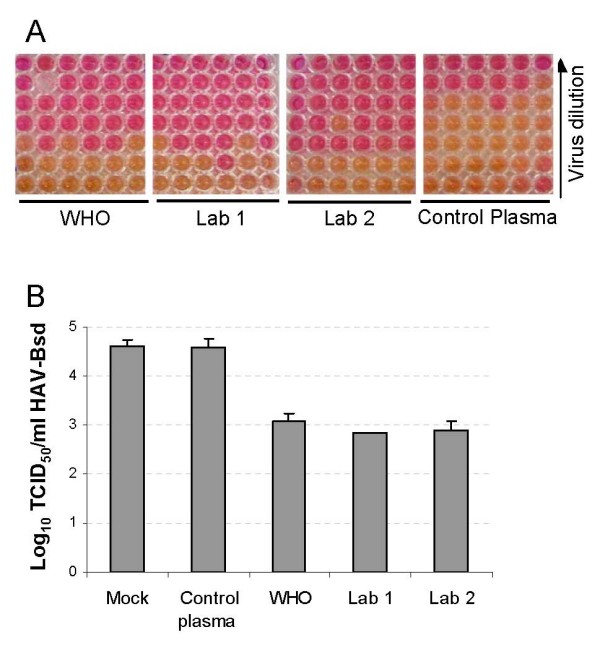
**Determination of the HAV neutralization potency of human IG preparations by ARTA**. HAV-Bsd was treated with 1% of Lab 1, Lab 2, WHO standard IG preparations, or negative plasma control, or mock-treated and titrated on 96-well plates containing Huh7-A-I cells. Four-fold serial dilutions of the neutralization reactions were inoculated in 6 wells per dilution. Complete medium with 2 μg/mL blasticidin was added to the wells, and the plates were incubated at 35°C for 8 days. (A) Color photograph of the 96-well plates obtained few minutes after taking the plates form the CO_2 _incubator. The mock-treated control plate is not shown, and one well of the WHO Standard is shown empty due to microbial contamination. (B) HAV-Bsd titers were determined by the Reed and Muench method counting positive (yellow and orange) and negative (pink and purple) wells. The ID50 program was used to calculate the viral titers as the log_10 _of tissue culture infectious dose 50% (TCID_50_) and standard deviations, which are shown as bars.

The HAV neutralization potency of the IG preparations was evaluated using the ARTA (Figure 4). To do so, neutralization reactions containing 1% of human IG preparations and 10^5 ^TCID_50 _of HAV-Bsd in 0.3 ml of complete cell culture media were incubated overnight at 4°C followed by 1 h incubation at 37°C. Four-fold dilutions of the neutralization reactions were titrated on 96-well plates containing Huh7-A-I cell monolayers. After 4 h adsorption at 35°C, cells were washed 3 times, and 0.2 ml of complete cell culture medium containing 2 μg/ml blasticidin was added per well. Plates were placed in a 35°C CO_2 _incubator for 8 days. The number of HAV-Bsd positive and negative wells was determined by visual examination of the orange-to-yellow and pink-to-purple wells, respectively (Panel A). Viral titers (Panel B) were calculated using the Reed and Muench method. The Lab 1, Lab 2, and WHO standard IG preparations neutralized approximately 1.5 log of HAV-Bsd. Treatment with the negative control plasma had no effect on the HAV-Bsd titer.

### Comparison of the ARTA and ELISA methods to evaluate anti-HAV neutralizing antibodies levels in IG preparations

**Figure 5:**
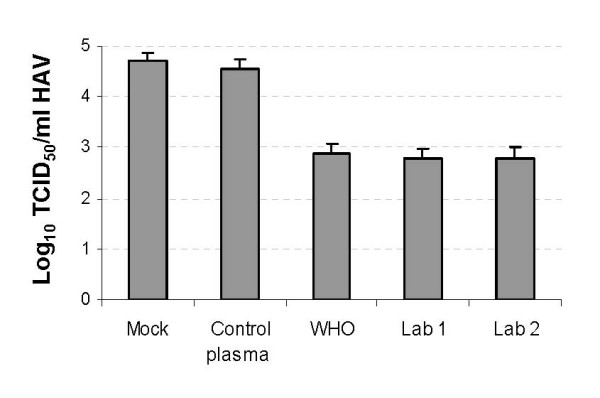
**Determination of the HAV neutralization potency of human IG preparations by ELISA**. HAV-Bsd was treated with 1% IG preparations as indicated in Figure 4 and titrated in 96-well plates containing Huh7-A-I cells. Four-fold serial dilutions of the neutralization reactions were inoculated in 8 wells per dilution and incubated in the absence of blasticidin for 2 weeks at 35°C. Cells were fixed and stained with anti-HAV mAb K2-4F2 and peroxidase-labeled goat anti-mouse antibodies. TMB substrate was added and color development was stopped by acidification. Absorbance at 450 nm was measured in an ELISA plate reader. Wells that developed at least 2 times the absorbance of mock-infected controls were considered positive for HAV-Bsd. Viral titers were calculated as in Figure 4.

In a parallel experiment, the same neutralization reactions used in the ARTA were titrated on 96-well plates containing Huh7-A-I cells. After viral absorption and washing, complete cell culture medium without blasticidin was added to the wells, and the plates were placed in a 35°C CO2 incubator for 14 days. Cells were fixed with 10% methanol and stained with an anti-HAV neutralizing monoclonal antibody and HRP-labelled goat anti-mouse secondary antibody. A TMB substrate was added to the wells, color development was stopped by acidification, and the plates were scanned in an ELISA plate reader. Wells that developed at least two times the absorbance of the uninfected control wells were considered positive for HAV-Bsd. Viral titers were calculated by the Reed and Muench method (Figure 5). The HAV-Bsd titers obtained by ARTA and ELISA were similar indicating that these titration systems are equivalent.

## Discussion

The ARTA method to titrate HAV is based on the antibiotic resistance of HAV-infected cells conferred by the blasticidin deaminase gene inserted into the virus genome. In the presence of blasticidin, HAV-Bsd-infected cells continue to metabolize and acidify the cell culture medium whereas uninfected cells die and the cell culture media turns pink to purple. At 8 days postinfection, there is a 100% correlation between the presence of surviving cells in the wells and the color of the cell culture medium, which obviates the use techniques of immunochemistry or molecular biology to detect the presence of the virus in the wells. Indeed, the HAV-Bsd titers determined by ARTA at 8 days post-infection and by ELISA at 2 weeks postinfection were almost identical (compare Figures 4 and 5) indicating that the ARTA and ELISA methods are equivalent. Since the Huh7-A-I cells are highly sensitive to low concentrations of blasticidin, the viral titers can also be determined at 5 days postinfection before the change in the color of the cell culture media becomes apparent by examination of the plates under the microscope (data not shown).

HAV is commonly titrated in monkey kidney or human fibroblast cell lines using attenuated strains of HAV that are highly adapted to grow in these cell cultures and contain a significant number of adapting mutations. For the ARTA, we selected a wild type based HAV vector and the human liver-derived Huh7-A-I cells to mimic as much as possible the HAV infection conditions found *in vivo*. Therefore, the ARTA described in this work is a better system to evaluate anti-HAV antibodies and screen for antivirals than other assays based on attenuated strains and non-hepatic cell substrates.

Human IG preparations are recommended for HAV pre- and post-exposure prophylaxis [11,12]. Although anti-HAV specific antibodies in the IG preparations for human use are evaluated by immunochemistry, an HAV neutralization test can provide a more direct evaluation of the potency of the IG preparations. We used the ARTA to evaluate the HAV neutralization potency of two commercially available IG preparations and a WHO anti-HAV standard. These three IG preparations contained similar levels of anti-HAV antibodies and had comparable neutralization potencies. Our data showed that the ARTA is a simple, rapid, and robust method to assess the HAV neutralization potency of human IG preparations. The ARTA described in this work reduces significantly the time, effort, and cost of HAV titrations and could be exploited for high throughput applications such as HAV epidemiological studies, screening of antivirals, and the routine assessment of the HAV neutralization potency of IG preparations for human use.

## Conclusion

The ARTA is a rapid and simple method to determine HAV titers by examining the color of the cell culture medium. HAV titers obtained by the ARTA and ELISA endpoint dilution assays are comparable. However, the ARTA requires shorter incubation times and no additional staining for viral antigens. We have successfully used the ARTA to evaluate the HAV neutralization potency of human IG preparations. The ARTA is an ideal assay to determine HAV titers for research purposes, and could also be used for large scale epidemiological studies, high throughput screening of antivirals, and the evaluation of anti-HAV antibodies in IG preparations for human use.

## Methods

### Cells and viruses

Huh7-A-I cells, a clone of human hepatoma Huh7 cells that support the stable growth of wt HAV in cell culture [24], were grown in Dulbeco's modified Eagle's medium supplemented with 10% fetal bovine serum (complete medium) at 37°C in a 5% CO_2 _incubator. The HAV-Bsd construct was derived from pHAV8Y-Bsd, which codes for the infectious cDNA of the wild type HM-175 strain of human HAV containing a A216V substitution at amino acid 216 of the 2B protein and a blasticidin resistance gene at the 2A-2B junction [24]. HAV-Bsd was grown in Huh7-A-I cells in the presence of 2 μg/ml blasticidin, and viral stocks were prepared by washing infected cell cultures and subjecting the cells to 3 freeze-and-thaw cycles.

### IG preparations

Stocks of commercially available liquid IG preparations, Immune Globulin (Human) and Immune Globulin Subcutaneous (Human), from two different Manufactures (Lab 1 and Lab 2) and the 1st WHO International Standard for Hepatitis A Immunoglobulin [27] containing 100 IU anti-HAV antibodies per ml were prepared as 16% IgG solutions. A stock of an anti-HAV antibody-negative human plasma donation prepared as a 1% IgG solution was used as negative control.

### Determination of anti-HAV antibodies in immune globulin preparations

IG preparations were diluted in complete medium and levels of anti-HAV antibodies were determined by the HAVAB EIA test (Abbott Laboratories) according to the manufacturer's instructions. Samples with OD values above the cutoff value were considered nonreactive, while those with OD values less than or equal to the cutoff value were considered reactive for anti-HAV antibodies. Samples were analyzed in duplicates, and the means and standard deviations of the OD from each dilution were calculated and plotted.

### Neutralization assay

Neutralization reactions in 0.3 ml of complete medium containing 1% IG preparations and 10^5 ^TCID_50 _HAV-Bsd were incubated rotating overnight at 4°C followed by 1 h at 37°C. The neutralization reactions were diluted in complete medium and titrated on 96-well plates containing confluent monolayers of Huh7-A-I cells.

### HAV titer determination by ARTA

Four- or ten-fold dilutions of the neutralization reaction in complete medium were titrated on 96-well plates containing confluent monolayers of Huh7-A-I cells. Four or six replica wells were inoculated per dilution. Plates were washed three times with serum free medium followed by the addition of 0.2 ml complete medium containing 2 μg/ml blasticidin per well. Plates were placed in a CO_2 _incubator at 35°C. After 8 days, plates were observed under the microscope, and wells containing cells were scored as positive. Alternatively, the plates were examined by the naked eye to determine the color of the cell culture media in each well. Orange and yellow wells were considered positive and pink and purple wells were considered negative. Viral titers were calculated using the Reed and Muench method [26].

### HAV titer determination by ELISA

The same neutralization reactions evaluated by ARTA were titrated in parallel by an endpoint dilution ELISA in 96-well plates containing Huh7-A-I cells but in the absence of blasticidin. Eight replica wells were inoculated per dilution, and plates were placed in a CO_2 _incubator at 35°C. Two weeks post-infection, HAV-Bsd titers were determined by ELISA [24]. Briefly, cells were fixed with 90% methanol, and stained with a 1:4,000 dilution of anti-HAV monoclonal antibody K2-4F2 (Commonwealth Labs), and a 1:5,000 dilution of peroxidase-labeled goat anti-mouse secondary antibody. TMB one-component peroxidase substrate (KPL Inc) was added to the wells, and the colorimetric reaction was stopped with 1% H_2_SO_4_. An increase in absorbance of at least two folds above the uninfected negative control wells was considered positive. Viral titers were determined by the method of Reed and Muench [26].

### Statistical analysis

Viral titers and standard deviations were calculated using the ID50 program developed by John L. Spouge (National Center for Biotechnology Information, NIH).

## Competing interests

The authors declare that they have no competing interests.

The findings and conclusions in this article have not been formally disseminated by the Food and Drug Administration and should not be construed to represent any Agency determination or policy."

## Authors' contributions

KK carried out the virology studies. KK performed the immunoassays with help from MLVT. KK and MLVT participated in the design of the study. MYY and GGK conceived of the study, and participated in its design and coordination. KK and GGK drafted the manuscript with the help of MLVT and MYY. All authors read and approved the final manuscript.
